# Castleman Disease: An Unexpected Cause of a Solitary Pleural Mass

**DOI:** 10.1155/2013/130515

**Published:** 2013-09-11

**Authors:** Fiachra Moloney, Maria Twomey, John Hinchion, Michael Maher

**Affiliations:** ^1^Department of Radiology, Cork University Hospital, Wilton, Cork, Ireland; ^2^Department of Cardiothoracic Surgery, Cork University Hospital, Wilton, Cork, Ireland

## Abstract

Castleman disease (CD) is a rare benign lymphoproliferative disorder, the etiology of which is unclear. Clinically it may manifest as localized disease (unicentric) or disseminated disease (multicentric). CD occurs in the thorax in 70% of cases, abdomen and pelvis in 15%, and in the neck in 10–15% of cases. We present a case of a pleural mass located posteriorly in a paraspinal location, which was discovered incidentally in a 50-year-old man and was subsequently resected followed by an unexpected diagnosis of Castleman disease on histological examination. In this report, we review the clinical and histological findings in a rare presentation of Castleman disease and discuss the findings in this case as part of an overall review of the typical radiological findings seen in Castleman disease.

## 1. Introduction

Castleman disease (CD), also known as angiofollicular lymph node hyperplasia or giant lymph node hyperplasia, is an uncommon nonmalignant lymphoproliferative disorder first described by Benjamin Castleman in 1954 [1]. It can occur at any age with a peak incidence in the third and fourth decades. CD occurs in the thorax in 70% of cases, abdomen and pelvis in 15%, and in the neck in 10–15% of cases [2]. 

Clinically, it may manifest as localized disease (unicentric) or widespread disease (multicentric) and histologically it is classified as hyaline vascular, plasmacytic, or mixed cellularity type disease. 

We report a rare presentation of unicentric Castleman disease, which presented as a pleural mass, located in a paraspinal location in an asymptomatic 50-year-old male patient.

## 2. Case Report

A 50-year-old male patient was found to have a mediastinal mass on a chest radiograph performed prior to surgical repair of a tibial fracture. He was asymptomatic and a nonsmoker. His past medical history was unremarkable. 

Physical examination of the cardiorespiratory system was normal. A full blood count and biochemical profile were normal.

**Figure 1:**
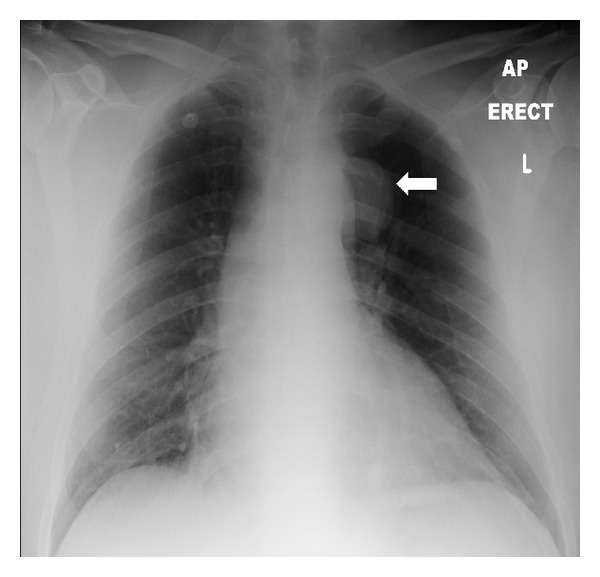
Chest radiograph in a 50-year-old male patient with Castleman disease. A soft tissue pleural-based mass is seen superior to the left hilum (arrow).

Chest radiograph demonstrated a 5 cm left-sided pleural or mediastinal mass (see Figure 1).

**Figure 2:**
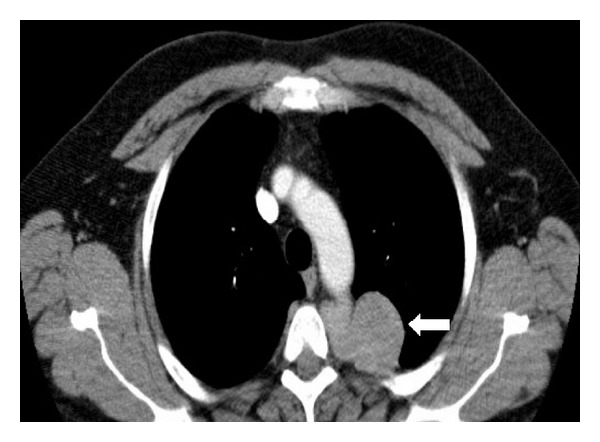
CT thorax with intravenous contrast in a 50-year-old male patient with Castleman disease. A 5 cm well-circumscribed, lobulated mass lesion with homogenously intense contrast enhancement is seen in the left posterior pleural space in a paraspinal location (arrow).

**Figure 3:**
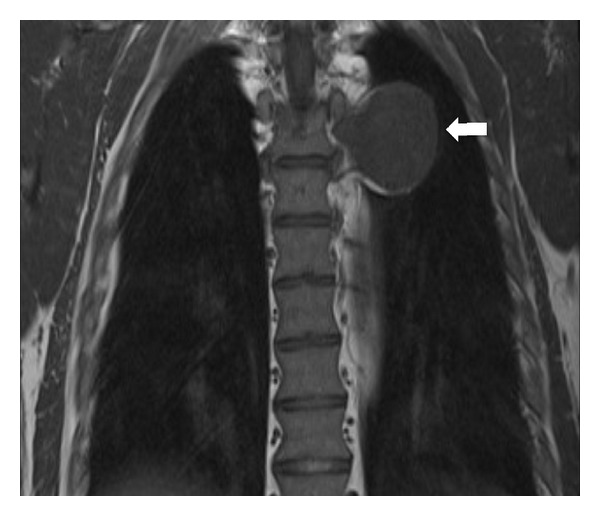
Chest MRI (coronal T1-weighted turbospin echo sequence) in a 50-year-old male patient with Castleman disease. The left paraspinal pleural-based mass is slightly hyperintense to skeletal muscle on T1-weighted sequences (arrow).

**Figure 4:**
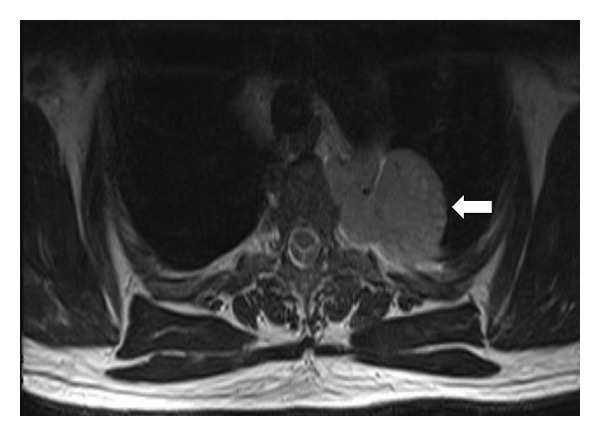
Chest MRI (axial T2 turbo spin echo sequence) in a 50-year-old male patient with Castleman disease. The left paraspinal pleural-based mass is heterogeneously hyperintense to skeletal muscle on T2-weighted sequences (arrow).

**Figure 5:**
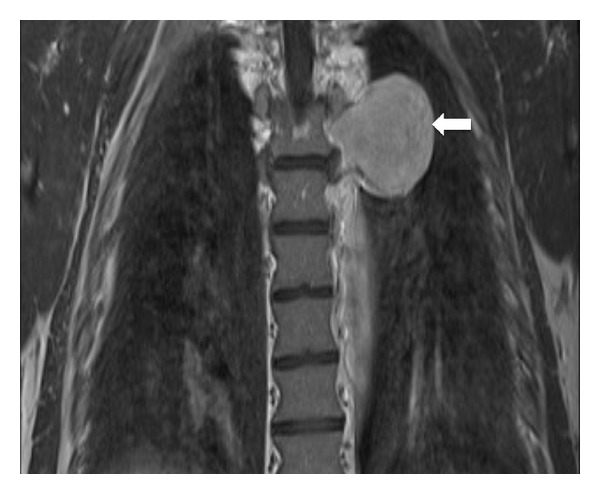
Chest MRI (coronal T1 gradient echo sequence with intravenous gadolinium contrast enhancement) in a 50-year-old male patient with Castleman disease. The left paraspinal pleural-based mass demonstrates homogenous enhancement following intravenous gadolinium administration (arrow).

A chest CT, with intravenous contrast, demonstrated a homogenously enhancing, well-circumscribed, lobulated pleural mass located posteriorly in a left paraspinal location adjacent to the descending thoracic aorta. There was no invasion of local tissues or widening of the adjacent neural foramina (see Figure 2). On magnetic resonance imaging (MRI), the lesion was found to be slightly hyperintense to skeletal muscle on T1-weighted MRI sequences and heterogeneously high in signal intensity on T2-weighted images (see Figures 3 and 4). There was avid homogenous contrast enhancement of the lesion following intravenous gadolinium administration (see Figure 5). A CT of the abdomen and pelvis was normal.

Differential diagnosis based on the constellation of imaging findings was mesothelioma, pulmonary hyalinizing granuloma, lymphoma, schwannoma, neurofibroma or ganglioneuroma. The patient proceeded to open thoracotomy and underwent uncomplicated primary excision. Histological examination revealed hyaline vascular variant unicentric Castleman disease. HIV testing was negative. The patient was referred to a hematologist for surveillance and remains asymptomatic 8 months following surgical excision.

## 3. Discussion

Castleman disease is a rare disease; the incidence of this lymphoproliferative disorder is uncertain and much of the available data is provided only by isolated case reports.

The etiology of the condition is unknown, but it has been described in association with a number of conditions including human immunodeficiency virus infection (HIV), human herpesvirus 8 infection (HHV-8), Kaposi's sarcoma, Hodgkin's lymphoma, non-Hodgkin's lymphoma, and syndrome (polyneuropathy, organomegaly, endocrinopathy, M protein and skin changes) POEMS [1]. 

Pathologically, there are two major histological subtypes: hyaline vascular subtype and plasmacytic; it may occasionally be a mixed subtype. Alternatively, it may be classified according to its biological behavior as unicentric or localized disease or multicentric. CD most commonly manifests as unicentric disease, which is invariably hyaline vascular type while multicentric disease is usually plasmacytic type [2, 3].

The majority of patients with unicentric disease are asymptomatic or may present with enlarged lymph nodes. Constitutional symptoms such as fever, night sweats, and weight loss are frequently seen in patients with multicentric disease.

Thoracic Castleman disease may present as a solitary, noninvasive mediastinal or hilar mass (50% of cases), a dominant mass with involvement of contiguous structures (40% of cases), or as a matted lymph node mass (10% of cases). 

Uncommonly, it may arise at other locations including the lung, pleura (as in this case), and pericardium. The key characteristic of unicentric Castleman disease is homogeneously intense contrast enhancement reflecting the hypervascularity of the lesion [4–6].

CD may atypically have a heterogeneous appearance on CT in the presence of internal calcification, necrosis, or fibrosis [7].

Multicentric thoracic CD typically demonstrates enhancing mediastinal and bihilar lymph nodes, thin-walled cysts, centrilobular nodules, ground-glass opacities, and bronchiectasis [4].

Multicentric abdominal disease is characterized by hepatosplenomegaly, diffuse lymphadenopathy, ascites, and thickening of the retroperitoneal fascia [8].

Lesions are typically slightly hyperintense to skeletal muscle on T1-weighted MRI sequences and heterogeneously hyperintense on T2-weighted images with avid homogenous enhancement following contrast administration [5].

Complete surgical excision of the involved lymph node is the treatment of choice in patients with unicentric CD and is usually curative.

Treatment options include debulking surgery and/or immunochemotherapy for multicentric disease, the outcomes of which are comparable [9]. CD of the pleura has been reported in previous reports to be associated with prominent pleural arteries and prominent adhesions to the adjacent lung [10]. These factors can impact surgical resection, resulting in significant blood loss and sometimes making VATS resection technically difficult or impossible. Patients with multicentric disease can occasional run a rapidly progressive course, which can be fatal.

## 4. Conclusion

Castleman disease is a rare condition and further research into its possible etiology and treatment, particularly of multicentric disease, is required. It is a great mimic of both benign and malignant conditions in the neck, thorax, and abdomen. It most commonly presents as an avidly enhancing lymph node mass in the thorax or abdomen. Although rare, the presence of avid enhancement within a thoracic lesion, even when located in the posterior mediastinum or pleura, should raise suspicion for CD. 

Previous reports have suggested that resection of CD masses can be difficult due to associated prominent vessels or adhesions, and appropriate highlighting of the possibility of CD preoperatively can facilitate appropriate preoperative planning.
